# Beyond 42 Days: A National Cohort Study of Maternal and Late Maternal Deaths in Brazil from 2010 to 2023

**DOI:** 10.3390/epidemiologia7040094

**Published:** 2026-07-07

**Authors:** Gustavo Gonçalves dos Santos, Elizabeth Mollard, Rita Pace Parascandalo, Sithokozile Maposa, Andrew Muriuki, Ricardo José Oliveira Mouta, Karina Franco Zihlmann, Carolliny Rossi de Faria Ichikawa, Cindy Ferreira Lima, Cesar Henrique Rodrigues Reis, Maria João Jacinto Guerra, Júlia Maria das Neves Carvalho, Ana Cristina Ribeiro da Fonseca Dias, Maria Luísa Santos Bettencourt, Cely de Oliveira, Bruna Feichas Renó, Eneida Tramontina Cerqueira, Katucha Rocha de Almeida Farias

**Affiliations:** 1 Programa de Pós-Graduação em Enfermagem, Grupo de Pesquisa Cuidar em Enfermagem na Saúde de Mulheres/Observatório de Inovações Tecnológicas na Saúde de Mulheres-Interseccionalidade, Raça e Gênero (GPCESM/OTIRG-Mulheres), Universidade Guarulhos (UnG), São Paulo 07023-070, SP, Brazil; 2 College of Nursing-Lincoln Division, University of Nebraska Medical Center (UNMC), Lincoln, NE 68588-0620, USA; elizabeth.mollard@unmc.edu; 3 Department of Midwifery, Faculty of Health Sciences, Università ta’ Malta (UM), 2080 Msida, Malta; rita.pace-parascandalo@um.edu.mt; 4 College of Nursing, University of Saskatchewan (USask), Saskatoon, SK S7N-2Z4, Canada; s.maposa@usask.ca; 5 Socially Responsible Evaluation in Education (SREed), University of Wisconsin-Milwaukee, Milwaukee, WI 53211, USA; ammuriuki13@gmail.com; 6 Departamento de Enfermagem Materno-Infantil, Faculdade de Enfermagem, Universidade do Estado do Rio de Janeiro (UERJ), Rio de Janeiro 20551-030, RJ, Brazil; ricardomoutta@gmail.com; 7 Departamento de Saúde, Educação e Sociedade, Universidade Federal de São Paulo-Campus Baixada Santista (UNIFESP/BS), São Paulo 11060-001, SP, Brazil; karina.zihlmann@unifesp.br; 8 Centro Universitário Faculdade de Medicina do ABC (FMABC), Santo André 09060-870, SP, Brazil; carolliny.ichikawa@fmabc.net; 9 Secretaria do Estado de Saúde de Minas Gerais, Organização Pan-Americana da Saúde (OPAS), Minas Gerais 31630903, MG, Brazil; cindy.lima@alumni.usp.br; 10 Programa de Pós-Graduação em Enfermagem, Universidade Federal de Mato Grosso do Sul (UFMS), Três Lagoas 79070-900, MS, Brazil; cesar.h@ufms.br; 11 Escola Superior de Enfermagem do Porto, Universidade do Porto (ESEP/UP), 4200-072 Porto, PT, Portugal; mjguerra@esenf.pt; 12 Escola Superior de Enfermagem de Coimbra, Universidade de Coimbra (ESEnfC/UC), 3046-851 Coimbra, PT, Portugal; juliacarvalho@ese.uc.pt; 13 Escola Superior de Saúde, Universidade dos Açores (ESS/UAc), 9500-315 Ponta Delgada, PT, Portugal; ana.cr.dias@uac.pt (A.C.R.d.F.D.); maria.ls.bettencourt@uac.pt (M.L.S.B.); 14 Universidade Santa Cecília (UNISANTA), Santos 11045-907, SP, Brazil; celyoliveira@unisanta.br (C.d.O.); brunareno@unisanta.br (B.F.R.); eneidatvc2017@gmail.com (E.T.C.); 15 Universidade Católica de Santos (UNISANTOS), Santos 11070-906, SP, Brazil; katucha.almeida@gmail.com

**Keywords:** maternal mortality, maternal deaths, mortality in pregnancy, childbirth and puerperium

## Abstract

Maternal mortality is a serious public health problem and reflects social, ethnic, racial, and regional inequalities in access to and quality of obstetric care. Despite advances in the surveillance and investigation of maternal deaths in Brazil, late maternal deaths (occurring between 43 days and 1 year after birth) are still underestimated and underexplored. Therefore, the objective of this study was to analyze the distribution and factors associated with maternal deaths and late maternal deaths in Brazil between 2010 and 2023. This was a population-based, retrospective cohort study with a quantitative approach, using secondary data from the Mortality Information System. All maternal deaths (Chapter XV of ICD-10) and late deaths recorded during the period were included. Sociodemographic, clinical, and administrative variables were analyzed. Statistical tests of association (chi-square, test of proportions, and 95% CI) were used, with a significance level of 5%. A total of 26,953 deaths were identified, of which 24,387 were maternal and 2566 were late deaths. Most deaths occurred among single, mixed-race women with 8 to 11 years of schooling, and residing in the Southeast region. Late deaths were more frequent in the South and among women aged 40 to 49. The main causes were direct obstetric conditions. A statistically significant association was observed between the type of death and sociodemographic variables. The results highlight structural inequalities in maternal mortality in Brazil and reinforce the importance of expanding postpartum surveillance beyond 42 days, with a focus on equity and continuity of care.

## 1. Introduction

Maternal mortality is an important indicator of public health and social inequality, reflecting not only the quality of obstetric care, but also the social and structural determinants that affect women of reproductive age. Globally, the incidence of maternal mortality varies widely. Some countries, such as Malta, report very low rates, with only two maternal deaths recorded between 2010 and 2023, one in 2010 and another in 2023 [1,2]. The World Health Organization (WHO) defines a maternal death as the death of a woman while pregnant or within 42 days of the termination of pregnancy, from any cause related to or aggravated by the pregnancy or its management, excluding accidental or incidental causes [3]. This 42-day threshold has long been used in surveillance systems and mortality estimates across countries.

Beyond maternal deaths, the concept of maternal near miss has emerged as an important indicator for evaluating the quality of maternal healthcare. The World Health Organization defines a maternal near miss as a woman who nearly died but survived a complication occurring during pregnancy, childbirth, or within 42 days after the termination of pregnancy. Because maternal near miss events occur more frequently than maternal deaths, they provide valuable opportunities to identify weaknesses in healthcare systems and factors associated with severe maternal outcomes. Together, maternal deaths, late maternal deaths, and maternal near miss cases represent a continuum of maternal health outcomes and contribute to a more comprehensive understanding of preventable maternal morbidity and mortality [1,2,3].

However, there is growing recognition that limiting maternal death surveillance to this early postpartum window may obscure a significant burden of mortality that occurs later. Deaths between 43 days and one year postpartum are classified by the WHO as late maternal deaths, yet these events are often underreported and underexamined in national datasets [4]. According to studies from high-income countries, including the United States and the United Kingdom, up to one half of pregnancy-related deaths occur after the 42-day threshold [5,6]. Many of these deaths are associated with cardiovascular, hypertensive, or mental health conditions that emerge or worsen during the extended postpartum period. Late maternal deaths are also less likely to be investigated, reported, or formally linked to the index pregnancy, which contributes to the underestimation of maternal mortality worldwide. Emerging global evidence suggests that late maternal deaths are not only more common than previously understood but may also reflect gaps in postpartum care, particularly for women with chronic conditions, psychosocial stress, or structural vulnerabilities [6,7].

Global variation in maternal mortality also highlights the influence of health system strength and social conditions. In Malta, for example, only two maternal deaths were recorded between 2010 and 2023, illustrating how rare such deaths can be under conditions of strong surveillance and comprehensive care [1,2]. By contrast, countries such as Brazil continue to experience elevated maternal mortality ratios and persistent disparities related to race, region, and education Although Brazil has expanded its maternal death surveillance and investigation systems over the past two decades, late maternal deaths remain poorly captured and insufficiently studied. Further, there is a need to deepen knowledge about the profile of women who die from causes related to pregnancy, childbirth and the puerperium, contributing to identifying racial, educational and regional inequalities throughout Brazil [8,9].

Maternal mortality is an important indicator of the quality of health systems. In Brazil, between 1990 and 2019, there was a reduction of approximately 49% in the maternal mortality ratio (MMR), falling from over 100 to approximately 50 deaths per 100,000 live births, according to data from the Global Burden of Disease (GBD) [10,11].

However, despite this progress, Brazil’s MMR remains above global targets. The GBD study highlighted that, although there has been a decrease in deaths related to hypertensive diseases, indirect causes, such as pre-existing conditions, have become responsible for the majority of cases in most states [10]. The COVID-19 pandemic further aggravated this situation. In 2020, the MMR jumped from 57 to 67 per 100,000 live births, and in 2021, it reached a staggering 107 deaths per 100,000, according to an analysis by Tenório et al. (2022) [12].

There are also striking regional and socio-ethnic disparities. Black women have almost twice the MMR compared to white women (125.8 vs. 64.1 per 100,000), and Indigenous women have up to 115 per 100,000, while the national average is around 68 per 100,000 [9]. Between 2010 and 2019, Brazil recorded an average of 5 late deaths per 100,000 births, with an estimated growth trend of 9.8% per year. Inequalities are evident across regions. In the South, the coefficient fell from 53.4 to 36.8 per 100,000 between 2000 and 2018, below the national average but still above the WHO target (<20). In the Northeast, since 2009, there has been a decline, although areas with high rates persist, such as Piauí and Maranhão [13,14].

This study aims to address that gap by analyzing maternal and late maternal deaths in Brazil from 2010 to 2023. We hypothesize that deaths occurring after the 42-day period differ significantly from early maternal deaths in terms of sociodemographic characteristics, underlying causes, and regional distribution. By comparing these two categories, we aim to improve understanding of late maternal mortality and to inform strategies for extending postpartum surveillance and care to better reach those at highest risk.

Given the above, the following questions arise: what are the sociodemographic and clinical characteristics associated with maternal deaths and late maternal deaths in Brazil between 2010 and 2023, and how do these occurrences differ from each other? The central hypothesis of this study is that maternal and late maternal deaths in Brazil do not occur homogeneously, but rather present significant differences in terms of sociodemographic (such as age, skin color, education, and marital status), clinical (such as direct or indirect obstetric causes), and regional (according to the area of residence) factors. Furthermore, it is assumed that most late maternal deaths occur after the 42-day postpartum surveillance period, highlighting the need to expand postpartum monitoring. Therefore, the present study aimed to analyze the distribution and factors associated with maternal deaths and late maternal deaths in Brazil between 2010 and 2023, identifying sociodemographic, clinical, and regional patterns.

## 2. Materials and Methods

### 2.1. Study Design

This is a population-based, retrospective cohort study with a quantitative approach, developed from the analysis of secondary records from the Sistema de Informação sobre Mortalidade (SIM), made available by the Departamento de Informática do Sistema Único de Saúde (DATASUS). The investigation was designed according to the recommendations of the Strengthening the Reporting of Observational Studies in Epidemiology (STROBE) for observational studies [15,16].

### 2.2. Study Location

The study covers the entire Brazilian territory, including the 27 federative units, and refers to the period between 1 January 2010 and 31 December 2023. The database used was obtained through the DATASUS website (http://tabnet.datasus.gov.br/cgi/tabcgi.exe?sim/cnv/mat10uf.def) accessed on 20 December 2025, which allows public access to microdata on maternal deaths recorded in SIM. The data were consulted based on the latest public update available until December 2024, including final records with a date of death up to the year 2023. All information refers to the place of residence of the deceased, and not to the place where the death occurred.

### 2.3. Study Population

The study population consisted of all Brazilian women whose deaths were recorded as maternal deaths (ICD-10 codes O00–O99) and late maternal deaths (occurring between 43 days and 1 year after the end of pregnancy), and recorded in the national SIM database during the period analyzed. The age range of women analyzed included all reproductive ages (10 to 49 years), with additional inclusions for cases outside this age range, according to the WHO maternal death criteria. All maternal deaths of Brazilian residents were included, regardless of age, as long as they met the World Health Organization definition criteria for maternal mortality. No sampling was applied, since the purpose of the study was to analyze all records available in the national SIM public database for the period in question. In sub-total, 24,387 maternal deaths and 2566 late maternal deaths were identified, totaling 26,953.

### 2.4. Study Variables

The variables analyzed were grouped according to sociodemographic, clinical and administrative categories: sociodemographic variables (age group, skin color, marital status, education level and region of residence); clinical variables (ICD-10 chapter and specific causes, type of obstetric cause (direct, indirect or unspecified maternal death) and time of death in relation to the pregnancy–puerperal cycle); administrative variables (status of death investigation (with summary record informed, without record, or not investigated); and the main outcome (occurrence of maternal death and late maternal death, as defined by SIM and ICD-10 (chapter XV: O00–O99) and related categories such as A34 and F53).

In cases where there was inconsistency between the declared cause of death (maternal or otherwise) and the time reported (pregnancy, childbirth, puerperium up to 42 days, puerperium from 43 days to less than 1 year or outside these periods), the type of underlying cause of death was adopted as the classification criterion, prioritizing it over the declared time. Despite advances, the fields in the Death Certificate that indicate the time of death (fields 43 and 44) still have a high percentage of non-completion or inconsistent completion, which may limit the analysis of late maternal deaths.

It is important to note that in 2011, there was a change in the Death Certificate model, with greater detailing of the variables collected. This year, both models (old and new) were used simultaneously, and there may be variations in the filling patterns. For additional details on this transition, it is recommended to consult the official document “Sistema de Informação sobre Mortalidade-SIM. Consolidation of the 2011 database”. In addition, on 1 April 2020, the Ministry of Health updated the SIM files related to deaths that occurred in 2019, which resulted in the change in the underlying cause of four records and the exclusion of one record.

For the purposes of this study, maternal deaths and late maternal deaths were classified according to the underlying cause-of-death coding registered in SIM and ICD-10. Late maternal deaths were defined as deaths coded as O96 or O97 and related categories recognized by the Brazilian mortality surveillance system. In situations where the reported timing of death and the underlying cause classification were inconsistent, the underlying cause of death was used as the primary criterion for classification. Therefore, some deaths reported as occurring between 43 days and less than one year postpartum remained classified as maternal deaths when the underlying cause did not meet the criteria for late maternal death according to ICD-10 coding.

### 2.5. Data Analysis

Initially, a descriptive analysis of the absolute and relative frequencies of maternal and late maternal deaths was performed according to the variables of interest. Proportions (%) were calculated in relation to the total number of maternal (n = 24,387) and late (n = 2566) deaths recorded in the period (total n = 26,953). The data were organized in tables according to ICD-10 categories, sociodemographic and administrative characteristics. Additionally, time trend analysis (such as Prais–Winsten regression) and calculation of maternal mortality ratios per 100,000 live births can be applied, through integration with data from the Sistema de Informação sobre Nascidos Vivos (SINASC). The main outcome analyzed was maternal death and late maternal death, as recorded in the SIM. Live birth data from SINASC were used only to calculate indicators such as the MMR, when applicable. All data were analyzed using electronic spreadsheets (Microsoft Excel^®^). The analyses were performed in R software (version 4.3) using the stats, epitools, dplyr and ggplot2 packages for organization, statistical analysis and data visualization [17].

The investigation of the central hypothesis of the study was conducted through descriptive and inferential analyses, focusing on the comparison between early and late maternal deaths according to sociodemographic and clinical variables. For statistical analysis, comparisons were made between maternal and late deaths according to sociodemographic variables, using different tests depending on the nature of the data. Pearson’s chi-square test (χ^2^) was used to verify the existence of an association between the type of death (maternal or late) and the categorical variables: age group, skin color, marital status, education level and region of residence. In cases where the expected frequencies in one or more cells were less than 5, as observed in the age group categories “10 to 14 years” and “50 to 59 years” and in the “indigenous” category of the skin color variable, Fisher’s exact test was applied, which is more appropriate for small samples. Additionally, proportion comparison tests (Z-test) were performed to assess, in a specific manner, statistically significant differences between the distribution of maternal and late deaths in the categories of each variable. For each comparison, the respective *p* values and 95% confidence intervals (95% CI) of the difference between the proportions were calculated. When necessary, categories with low frequency were regrouped in order to meet the assumptions of the tests applied. All statistical analyses considered a significance level of 5% (α = 0.05).

### 2.6. Ethical Aspects

This study used secondary data from the public domain, available without identifying the subjects by name, ensuring the anonymity of the information. In accordance with Resolution No. 510/2016 of the National Health Council, which waives the need for ethical assessment for studies with public and anonymous databases, this study is exempt from submission to the Research Ethics Committee [18]. Even so, all stages of the study were conducted in compliance with the ethical and legal principles in force for health research in Brazil.

## 3. Results

The highest proportion of maternal deaths occurred among women aged 30 to 39 (40.8%), followed by those aged 20 to 29 (38.8%). These two age groups accounted for almost 80% of deaths, which reflects the period of greatest fertility for women. Adolescents aged 15 to 19 accounted for 11.2% of deaths, while girls aged 10 to 14 accounted for 0.8%, standing out as a particularly vulnerable group. In the 40 to 49 age group, mortality was 8.3%, but proportionally, they accounted for 10.1% of late deaths, suggesting a higher risk of long-term complications. Women aged 50 or over barely appear in the records.

Most maternal deaths occurred among brown women (52.1%), followed by white women (31.8%) and black women (11.1%). Indigenous and Asian women accounted for 1.6% and 0.2%, respectively. This distribution indicates significant racial inequality, especially when considering the proportional underrepresentation of white women among late deaths (35.1%) in relation to brown women (47.2%). Black women (black and brown women combined) account for more than 63% of maternal deaths and approximately 61.7% of late deaths, highlighting structural inequalities in access, quality of care and health outcomes.

Single women accounted for the majority of deaths, accounting for 48.3% of maternal deaths and 53.7% of late deaths. Married women were the second most affected group (28.4% and 26%, respectively). This data suggests that social and family support may be related to the outcomes, or that marital status is a marker of other socioeconomic vulnerabilities. It is also worth noting the significant proportion of missing data (7.4% and 6.3%), which limits a more precise analysis.

Most maternal deaths occurred among women with 8 to 11 years of schooling (39%), followed by those with 4 to 7 years (22.9%). Women with higher education or more accounted for only 11.5% of deaths, indicating that higher education tends to be a protective factor. On the other hand, 15.2% of the information on education was missing, which compromises part of the analysis. Late deaths followed a similar trend, with a predominance among women with 8 to 11 years of schooling (39.2%) and 4 to 7 years (25.9%).

Maternal deaths were concentrated in the Southeast region (35.3%), followed by the Northeast (32.6%) and North (13.7%). However, when we look at late deaths, there is a relative increase in the South (18.4%), suggesting possible regional differences in the quality of care and postpartum surveillance. The Southeast maintained the highest absolute number of late deaths (37.8%), possibly reflecting the greater number of births and/or better reporting. The North, although representing 13.7% of maternal deaths, accounts for only 8.2% of late deaths, which may indicate underreporting or less tracking of late causes, as shown in the Table 1.

Analysis of data on the causes and characteristics of maternal deaths according to the International Classification of Diseases–10th Revision (ICD-10) shows that almost all deaths are directly related to the pregnancy–puerperal cycle. Of the 24,387 maternal deaths recorded, 98.7% (24,058) were classified in Chapter XV of ICD-10, which covers direct obstetric causes, while only 1.3% (305) were distributed among chapters of indirect causes, such as infectious diseases, mental disorders and neoplasms. Among these, HIV infection was the main cause identified, responsible for 304 deaths (1.2%).

When detailing the specific causes within the obstetric chapter, it is observed that “other obstetric conditions not elsewhere classified” (NCOP) lead with 35.1% of deaths (8564), followed by hypertensive disorders of pregnancy (19.6%), complications of labor and delivery (14.4%) and complications related to the puerperium (12.6%). Abortion was the cause of 1785 deaths (7.3%), standing out as an important public health problem, especially considering the context of legal restrictions and barriers to access to safe services. Notably, only 1 death was classified directly under the code “childbirth”, which suggests that complications are usually related to events secondary to labor itself.

Regarding the time of death, most maternal deaths occurred in the puerperium up to 42 days after delivery (57.3%), followed by deaths during pregnancy, delivery or abortion (29.4%). Late deaths (from 43 days to less than 1 year after delivery) accounted for only 3.9% of maternal deaths, but represented 93.5% of late deaths (2400 of 2566). This data highlights the importance of expanding surveillance beyond the 42 days of the puerperium, especially considering the prolonged risk of maternal morbidity and mortality.

Analysis of the type of maternal death shows a predominance of direct obstetric causes (61.6%), such as hemorrhages, puerperal infections and hypertensive disorders, while indirect obstetric causes, associated with pre-existing diseases or diseases acquired during pregnancy that are aggravated by the course of pregnancy (such as infections and cardiovascular diseases), accounted for 35.3%. In 3.1% of cases, the nature of death was not specified, indicating a limitation in the quality of the information recorded.

Regarding the status of death investigations, it was observed that 90.8% of maternal deaths were investigated with a summary record, demonstrating broad surveillance coverage. However, 6.8% of cases were not investigated, which represents a significant weakness in determining the causes and circumstances of these deaths. Regarding late deaths, the percentage of investigations is even higher: 93.6% had a summary record, reflecting the growing attention of obstetric surveillance to this type of occurrence, as shown in Table 2.

The analysis of the association between the type of death (maternal vs. late) and the sociodemographic variables revealed statistically significant results for all categories analyzed. Regarding the age group, a *p*-value equal to 0.00085 was identified, indicating a significant association with the type of death. The association between the type of death and the age group, considering the groups grouped as 10 to 19 years, 20 to 29 years, 30 to 39 years and 40 years or older, presented a *p*-value of 0.0025. The minimum expected value in the cells was 21,910, all above the critical limit of 5, which validates the test applied. Thus, there was a statistically significant association between the grouped age group and the type of death (maternal vs. late). Regarding skin color, the association with the type of death was also significant, with a minimum expected value of 5.8, which ensures the statistical validity of the test applied. Therefore, the Chi-square was considered adequate and the results reliable, indicating a statistically significant result. This finding demonstrates that the distribution of maternal and late deaths differs significantly between indigenous and white women, even considering the reduced number of cases in the indigenous population.

For the education variable, the Chi-square test resulted in a *p*-value, with a minimum expected value of 59.88, which confirms the robustness of the test applied and the existence of a significant association between the level of education and the type of death. Finally, the region of residence presented a highly significant association (*p* < 10^−49^), with a minimum expected value of 21,573, indicating a strong relationship between the place of residence and the distribution of maternal and late deaths, as shown in Table 3.

The comparison was performed by means of the difference in proportions, with calculation of the 95% confidence interval and the *p*-value, with the aim of verifying whether these proportions differ statistically between the two groups. While most deaths occurred within the first 42 days postpartum, late maternal deaths accounted for 9.5% of all maternal deaths analyzed. Among deaths classified as late maternal deaths, the vast majority occurred between 43 days and less than one year after delivery, consistent with the ICD-10 definition of late maternal mortality. This difference was statistically significant (*p* < 0.001), with a 95% CI for the difference in proportions ranging from −0.907 to −0.887, evidencing the predominance of late deaths in the immediate post-puerperal period. In addition, categories such as direct obstetric death (61.6%) and indirect obstetric death (35.3%) appeared only among total maternal deaths, with statistically significant differences (*p* < 0.001).

Regarding the variable “investigation status”, although most deaths in both groups were investigated with a summary provided (90.8% of total deaths and 93.6% of late deaths), there was a significant difference (difference of −2.8; 95% CI [−0.038, −0.018]; *p* < 0.001), suggesting a higher percentage of formal investigation among late deaths. Another relevant point is that, even in categories of lower occurrence such as “inconsistent period reported” or “death not investigated”, significant differences were found between the groups. The only category with no statistical difference was “investigated without summary available” (*p* = 0.7275), as shown in Table 4.

## 4. Discussion

This study analyzed national data on maternal and late maternal deaths in Brazil between 2010 and 2023. While most maternal deaths occurred within the first 42 days postpartum, over half were classified as late maternal deaths, occurring between 43 days and one year after delivery. This finding alone challenges the adequacy of current surveillance definitions for maternal death and suggests that important risk periods extend well beyond the early puerperium.

We found that women between the ages of 20 and 39 accounted for nearly 80% of all maternal deaths. Nevertheless, a substantial proportion of deaths occurred at the extremes of reproductive age, with adolescents aged 15–19 accounting for 11.2% of deaths and women aged 40–49 representing 8.3%. These findings reinforce earlier studies demonstrating higher risk among women over 30, particularly those over 40, where complications such as hypertensive disorders and coagulopathies are more prevalent [8,19,20].

Regarding the ethnoracial profile, the findings of this study corroborate recent literature that points to the persistence of structural racism in the production of health inequalities. Black and brown women have less access to quality obstetric care, fewer prenatal appointments, and a higher frequency of adverse outcomes, including maternal and neonatal death [8]. A study of more than 21 million live births between 2012 and 2022 found that skin color was a determining factor in adverse maternal outcomes, independent of socioeconomic factors [9].

Racial and ethnic disparities were also prominent. More than 63% of deaths occurred among women identified as brown or black, consistent with national evidence showing that Black women face nearly twice the maternal mortality risk of their White counterparts across all regions and age groups [9]. There were educational disparities noted with 39% of maternal deaths occurring among women with incomplete secondary education, compared to just 11.5% among women with a college degree. Lower educational attainment has been consistently associated with reduced access to prenatal care and delayed identification of complications [21].

Social context appeared to play a substantial role as well. Nearly half of all maternal deaths occurred among single women (48.3%). This aligns with findings from Recife, where 60% of women who died had no partner, suggesting that lack of social and emotional support may hinder health-seeking behaviors and continuity of care [9]. Another relevant finding is the association between low education and maternal death. Women with up to seven years of schooling had a higher proportion of deaths, which reinforces the role of education as a protective factor. Women with higher education tend to access health services earlier and understand the risks associated with pregnancy and the postpartum period [21].

The observed associations between maternal mortality and factors such as race, education, marital status, and region of residence should also be interpreted through the lens of social justice and structural discrimination. Maternal mortality is not merely a clinical outcome but a manifestation of broader social inequities that shape women’s opportunities to access healthcare, education, employment, housing, and social protection. Women belonging to historically marginalized racial and ethnic groups, those with lower educational attainment, and those living in socioeconomically disadvantaged regions are disproportionately exposed to structural barriers that increase their vulnerability during pregnancy and the postpartum period. These disparities reflect the cumulative effects of structural racism, gender inequality, and social exclusion, which influence both health status and access to timely, high-quality maternal care. From a reproductive justice perspective, reducing maternal mortality requires not only improvements in healthcare services but also policies aimed at addressing the social determinants of health, promoting equity, and eliminating discriminatory practices within health systems and society. Therefore, maternal mortality should be understood as both a public health challenge and a social justice issue [7,8,9,19,21,22].

Another study on maternal mortality showed in its results an 11.9% increase in the number of maternal deaths in Brazil and in the country’s Mortality Rate, from 52.29 to 65.13 maternal deaths per 100,000 live births. The main causes of maternal deaths were: other maternal illnesses that complicate pregnancy, childbirth, and the puerperium (17.1%); eclampsia (11.8%); gestational hypertension with significant proteinuria (6.2%); postpartum hemorrhage (5.8%); puerperal infection (5.1%) and premature placental abruption (4.2%). There was a higher number of maternal deaths in women with 4 to 7 years of schooling (23.8%), of mixed race/color (42.7%), with single marital status (53%) and aged 20 to 29 (41.8%). In these results, the place of death was predominantly the Hospital (91.2%).

The regional inequalities identified in this study also reflect historical disparities in the organization of health systems in Brazil. The higher proportion of maternal deaths in the Southeast may reflect both the larger population and better reporting. However, the increase in late deaths in the South highlights potential gaps in the continuity of postpartum care in this region. A time-series study identified a significant increase in late maternal mortality between 2015 and 2021 in the South and Northeast regions, especially in peripheral urban areas [13].

Late maternal deaths were also disproportionately high in the South region (18.4%), indicating possible deficiencies in long-term follow-up and postnatal monitoring. Previous studies have noted a sharp increase in late maternal deaths in the South during the 2020–2021 period [22]. Thus, these regional differences may also reflect the broader impact of the COVID-19 pandemic, which disproportionately affected maternal health systems in Brazil. Between 2020 and 2021, Brazil reported some of the highest maternal mortality rates in the world related to COVID-19 complications, many of which occurred in the late postpartum period and were often underreported. Structural barriers to care, hospital overload, and disruptions to prenatal and postpartum services likely exacerbated existing racial, regional, and socioeconomic disparities during this time [5,23].

From a clinical perspective, 98.7% of deaths were due to direct obstetric causes. Non-classified obstetric complications (NCOP) accounted for 35% of deaths, while hypertensive disorders made up 19.6%. Hemorrhage, postpartum infections, and abortion-related complications comprised another 7.3%. These distributions are consistent with earlier findings highlighting the elevated risk of these conditions among Black, Indigenous, and older women [9,20,24,25]. However, the remarkably high proportion of deaths labeled as direct causes may reflect limitations in death certificate data and the potential underrecognition of indirect causes, such as chronic illnesses, psychiatric conditions, or substance use. In high-surveillance settings such as the United States and United Kingdom, comprehensive maternal death reviews have revealed that a substantial proportion of late maternal deaths stem from cardiovascular disease, suicide, and drug overdose [5]. These findings suggest that improved identification of indirect and late causes could further clarify the burden and timing of maternal mortality in Brazil.

Although data indicate a predominance of direct obstetric causes, it is important to highlight that maternal deaths from indirect causes, such as cardiovascular and respiratory diseases, and mental disorders, are increasing, especially in late pregnancy. International studies show that more than half of maternal deaths in the United States between 2017 and 2019 were caused by preexisting conditions exacerbated by pregnancy, including heart disease, hemorrhage, and mental health disorders, especially in the late postpartum period [23]. In the United States, recent data reinforce this trend, indicating that indirect causes have increased proportionally in recent years, particularly among Black women and those with low levels of education [26].

Another relevant point is the underreporting of maternal deaths in the late period, which compromises surveillance and public policy development. An analysis of maternal mortality committees in the US showed that 84% of pregnancy-related deaths were preventable, implying systemic failures in care in both high- and middle-income countries [23]. In Brazil, the lack of standardization in coding causes of death, coupled with incomplete data in the SIM, limits a full understanding of the profile of late deaths and their association with social and structural determinants [11].

The COVID-19 pandemic has further exacerbated these vulnerabilities. Brazil had one of the highest absolute numbers of COVID-19-related maternal deaths between 2020 and 2022, with the majority occurring in the late postpartum period, often outside of a hospital setting. The overload of health services, the discontinuity of prenatal care, and the shortage of obstetric and intensive care beds contributed to the increase in preventable deaths. These data reinforce the urgency of expanding maternal surveillance beyond 42 days postpartum, incorporating active monitoring up to one year after birth, as already recommended by international organizations [12,22].

International comparisons highlight that maternal mortality remains substantially higher in Brazil than in many high-income countries. Nations such as Spain, Italy, Portugal, and Canada report maternal mortality ratios below 10 deaths per 100,000 live births, largely attributed to universal healthcare coverage, robust maternal surveillance systems, and continuity of care throughout pregnancy and the postpartum period [26,27,28,29,30]. Despite these favorable indicators, studies from these countries continue to identify disparities among socially vulnerable populations, including ethnic minorities, migrants, and women living in geographically isolated areas [27,28,29,30,31]. Similarly, our findings demonstrate that maternal mortality in Brazil is strongly influenced by social, racial, educational, and regional inequalities. These results suggest that, although healthcare system organization plays an important role, reducing maternal mortality also requires addressing structural determinants of health and ensuring equitable access to high-quality maternal care across all population groups.

Soares et al., 2013 [32], showed that in the first three-year period, in referral hospitals for high-risk pregnancies, the hospital fatality rate was 158.4/1,000,000 deliveries and, in the second, 132.5/1,000,000, and the main causes were: pre-eclampsia/eclampsia, urinary tract infection, puerperal infection, and indirect causes. In referral hospitals for low-risk pregnancies, the hospital fatality rates were 7.2/100,000 and 80/100,000, and the main causes were: hemorrhages, embolisms, and anesthetic complications. In total, 90% of deaths in the second three-year period were considered preventable and it was concluded that there are difficulties in treating obstetric complications at both low and high complexity levels of care and that the training of professionals to handle obstetric emergencies and monitoring the use of protocols at all hospital levels should be prioritized to reduce preventable maternal deaths [32].

Dias et al., 2015 [33], point out that the Southeast and Northeast regions concentrate the highest number of maternal deaths. Of the 1719 maternal deaths reported in 2010, 604 occurred in the Southeast region and 598 in the Northeast region. In the North, there were 192, in the South 193, and in the Central-West, 132, according to the DATASUS report [33]. They also highlight that in Brazil, direct obstetric causes account for 66.7% of maternal deaths and their main causes are: hypertensive diseases, hemorrhagic syndromes, complications of childbirth and puerperal infections, being closely related to socioeconomic factors.

Although the deaths of women during the pregnancy–puerperal cycle are associated with the unsatisfactory provision of health care and reflect the inadequate quality of care, it is an important indicator of poverty, social inequality, coverage and quality of care for the health needs of the population [34]. Particularly important is the importance of paying special attention to monitoring women during the late postpartum period, given the potential positive impact on preventing these preventable deaths. Therefore, it is recommended that future research incorporate an in-depth analysis of the underlying causes of late maternal mortality, in addition to interventions in professional training and improving the quality of health services [13].

An additional challenge in the interpretation of late maternal mortality concerns the attribution of causality between pregnancy and death. While ICD-10 and ICD-MM provide standardized criteria for classifying maternal and late maternal deaths, determining whether a death occurring months after delivery is directly or indirectly related to pregnancy is not always straightforward. Women with preexisting chronic conditions, such as HIV infection, malignancies, cardiovascular diseases, or severe mental health disorders, may die during the late postpartum period from causes that were present before pregnancy but potentially aggravated by physiological changes associated with gestation and childbirth. In such cases, distinguishing between deaths caused by pregnancy, deaths indirectly influenced by pregnancy, and deaths merely coincidental to pregnancy may be challenging. This ambiguity highlights the importance of comprehensive clinical review processes, maternal mortality committees, and detailed case investigations to improve the accuracy of classification and interpretation of late maternal mortality statistics [3,5,10,11,23].

Although countries such as Brazil and Mexico continue to report maternal mortality ratios above the targets proposed by the Sustainable Development Goals, important reductions have been observed over the last decades. These improvements have been attributed to the expansion of universal health coverage, increased access to prenatal care, greater availability of skilled birth attendants, improvements in emergency obstetric care, and the strengthening of maternal death surveillance systems. In Brazil, the implementation of public policies through the Unified Health System (SUS), the expansion of primary healthcare coverage through the Family Health Strategy, and the establishment of Maternal Mortality Committees have contributed to earlier identification of obstetric complications and improvements in care quality. Similarly, Mexico has reported reductions associated with expanded institutional childbirth, improved referral systems, and broader access to reproductive health services. Nevertheless, persistent social, racial, ethnic, and regional inequalities continue to limit further progress, explaining why maternal mortality levels remain higher than those observed in high-income countries despite the overall downward trend.

Despite the robust national scope, this study has several limitations. The use of secondary data from the SIM, while comprehensive, is susceptible to underreporting, missing data, and delays in record consolidation. Key fields such as time of death relative to the pregnancy and sociodemographic variables like education and marital status were inconsistently completed, which may limit the precision of our classification. Moreover, the reliance on death certificate coding may obscure indirect causes of death, particularly when chronic conditions aggravated by pregnancy are underrecognized. The absence of clinical variables such as prenatal visit counts, delivery type, and access to specialized care also limits our ability to fully understand the care trajectories leading to maternal death. Future research could benefit from the application of decomposition methods, such as the Blinder–Oaxaca decomposition, to quantify the relative contribution of observable sociodemographic factors and unobserved structural determinants to ethnic and regional disparities in maternal and late maternal mortality. Such approaches may help disentangle the role of education, socioeconomic conditions, healthcare access, and structural discrimination in shaping maternal health outcomes.

An additional limitation concerns the accuracy of cause-of-death coding and certification practices. Maternal and late maternal mortality estimates are highly dependent on the quality of information recorded on death certificates and the correct application of ICD-10 codes by physicians responsible for certifying deaths. Inadequate recognition of pregnancy-related conditions, incomplete documentation of the pregnancy status, or errors in coding may result in both underreporting and overreporting of maternal deaths. This challenge is particularly relevant for late maternal deaths, which occur several weeks or months after delivery and may not be readily linked to the preceding pregnancy. Consequently, some deaths may be incorrectly classified as non-maternal, while others may be attributed to maternal causes without sufficient clinical evidence. These limitations highlight the importance of continuous professional training, standardized certification procedures, and rigorous mortality surveillance systems to improve the accuracy of maternal mortality statistics.

Nevertheless, our findings provide strong support for revisiting Brazil’s postpartum surveillance frameworks. Recognizing and recording late maternal deaths, particularly among socially marginalized populations, could help uncover system-level failures that remain invisible under current reporting standards. Extending the monitoring window to one year postpartum would align Brazil with WHO recommendations and international best practices and would generate actionable data to guide interventions. This study also highlights the need for standardized, complete, and accurate documentation of maternal deaths, particularly regarding cause, timing, and social determinants.

## 5. Conclusions

This study showed that maternal and late maternal deaths in Brazil present distinct and unequal patterns in terms of sociodemographic, regional, and clinical factors. The data reveal that maternal mortality in Brazil continues to be a reflection of structural inequalities, race, education, social support, region, age and class. These disparities were further intensified during the COVID-19 pandemic, which strained health systems and disproportionately affected vulnerable populations. Addressing these challenges will require both clinical and policy-level interventions. Expanding postpartum surveillance beyond the first 42 days is an important step not only to safeguard maternal health, but also to improve data accuracy and policy response. Targeted efforts are needed to monitor women at the extremes of reproductive age, promote racial equity through anti-racist training of health professionals, and implement social policies that improve education, income, and access to healthcare.

In addition, the development and adoption of extended maternal care protocols, particularly those addressing hypertensive conditions, mental health, and psychosocial support, should be prioritized to reduce preventable late deaths. Public policies aimed at reducing maternal mortality must confront these structural inequities and invest in continuous postpartum care throughout the first year after birth, with focused attention on the most at-risk populations.

## Figures and Tables

**Table 1:** Distribution of maternal and late maternal deaths according to sociodemographic characteristics (n = 26,953). Brazil 2010–2023.

Variable	Maternal Deaths (n)	% Maternal Deaths	Late Deaths (n)	% Late Deaths
**Age**				
10 to 14 years	202	0.8%	10	0.4%
15 to 19 years	2730	11.2%	256	10%
20 to 29 years	9466	38.8%	1023	39.9%
30 to 39 years	9942	40.8%	1018	39.7%
40 to 49 years	2017	8.3%	258	10.1%
50 to 59 years	27	0.1%	-	-
Ignored	3	0%	1	0%
**Skin color**				
White	7768	31.8%	902	35.1%
Black	2711	11.1%	373	14.5%
Yellow	59	0.2%	2	0.1%
Brown	12,710	52.1%	1212	47.2%
Indigenous	392	1.6%	18	0.7%
Ignored	747	3.1%	59	2.3%
**Marital status**				
Single	11,796	48.3%	1379	53.7%
Married	6923	28.4%	667	26%
Widow	143	0.6%	18	0.7%
Legally separated	450	1.8%	64	2.5%
Other	3260	13.4%	277	10.8%
Ignored	1815	7.4%	161	6.3%
**Education**				
None	589	2.4%	40	1.6%
1 to 3 years	2197	9%	248	9.7%
4 to 7 years	5593	22.9%	664	25.9%
8 to 11 years	9516	39%	1005	39.2%
12 years and over	2796	11.5%	251	9.8%
Ignored	3696	15.2%	358	13.9%
**Region of residence**				
North	3349	13.7%	211	8.2%
North East	7944	32.6%	730	28.5%
Southeast	8612	35.3%	969	37.8%
South	2400	9.8%	472	18.4%
Midwest	2082	8.5%	184	7.2%
**Total**	24,387	100%	2566	100%

**Source:** Prepared by the authors. Brazil, 2025.

**Table 2:** Maternal and late maternal deaths according to clinical causes and investigation (n = 26,953). Brazil 2010–2023.

Variable	Maternal Deaths n (%)	Late Maternal Deaths n (%)
**ICD-10 Chapter**		
I. Certain infectious and parasitic diseases	35 (0.1%)	0 (0%)
II. Neoplasms	6 (0%)	0 (0%)
V. Mental and behavioral disorders	18 (0.1%)	0 (0%)
XV. Pregnancy, childbirth and puerperium	24,058 (98.7%)	2566 (100%)
**Specific causes (ICD-10)**		
Other bacterial diseases	1 (0%)	0 (0%)
Human immunodeficiency disease	304 (1.2%)	0 (0%)
Neoplasm of uncertain or unknown behavior	6 (0%)	0 (0%)
Behavioral syndromes associated with physiological dysfunction and physical factors	18 (0.1%)	0 (0%)
Pregnancy ending in miscarriage	1785 (7.3%)	0 (0%)
Edema, proteinuria and hypertensive disorders in pregnancy	4777 (19.6%)	0 (0%)
Other maternal disorders predominantly related to pregnancy	1000 (4.1%)	0 (0%)
Maternal care related to fetus, amniotic cavity and possible delivery problems	1354 (5.6%)	0 (0%)
Complications of labor and delivery	3502 (14.4%)	0 (0%)
Childbirth	1 (0%)	0 (0%)
Complications predominantly related to the puerperium	3075 (12.6%)	0 (0%)
Other obstetric conditions (NCOP)	8564 (35.1%)	2566 (100%)
**Time of death**		
During pregnancy, childbirth or abortion	7179 (29.4%)	4 (0.2%)
During the puerperium, up to 42 days	13,969 (57.3%)	51 (2%)
During the puerperium, from 43 days to less than 1 year	942 (3.9%)	2400 (93.5%)
Not during pregnancy or postpartum period	427 (1.8%)	62 (2.4%)
Inconsistent reported period	176 (0.7)	0 (0%)
Not informed or ignored	1694 (6.9)	49 (1.9%)
**Type of maternal death**		
Direct obstetric maternal death	15,032 (61.6%)	0 (0%)
Indirect obstetric maternal death	8604 (35.3%)	0 (0%)
Unspecified obstetric maternal death	747 (3.1%)	0 (0%)
**Investigation status**		
Death investigated, with summary record provided	22,152 (90.8%)	2402 (93.6%)
Death investigated, without summary record provided	572 (2.3%)	63 (2.5%)
Death not investigated	1663 (6.8%)	101 (3.9%)
**Total**	24,387 (100%)	2566 (100%)

**Source:** Prepared by the authors. Brazil, 2025.

**Table 3:** Association tests between type of death and sociodemographic variables (n = 26,953). Brazil 2010–2023.

Variable	IC 95%	*p*-Value
**Age**		
10 to 14 years	-	-
15 to 19 years	0.002–0.027	0.057
20 to 29 years	0.012–0.041	<0.001
30 to 39 years	0.010–0.037	<0.001
40 to 49 years	0.005–0.018	0.008
50 to 59 years		
Ignored		
**Skin color**		
White	-	-
Black	0.009–0.021	<0.001
Yellow	0.002–0.001	-
Brown	0.038–0.012	<0.001
Indigenous	0.002–0.012	<0.001
Ignored		
**Marital status**		
Single	0.015–0.034	<0.001
Married	0.017–0.009	<0.001
Widow	0.002–0.012	-
Legally separated	0.002–0.001	-
Other	0.002–0.008	-
Ignored		
**Education**		
None	0.001–0.007	<0.05
1 to 3 years	0.002–0.011	<0.05
4 to 7 years	0.006–0.018	<0.01
8 to 11 years	0.032–0.012	<0.001
12 years and over	0.004–0.002	-
Ignored		
**Region of residence**		
North	0.025–0.011	<0.001
North East	0.019–0.008	<0.001
Southeast	-	-
South	0.012–0.026	<0.001
Midwest	0.003–0.005	-

**Source:** Prepared by the authors. Brazil, 2025.

**Table 4:** Association tests categorization ratios between total and late maternal deaths (n = 26,953). Brazil 2010–2023.

Variable	IC 95%	*p*-Value
**Time of death**		
During pregnancy, childbirth or abortion	0.287–0.299	<0.001
During the puerperium, up to 42 days	0.545–0.561	<0.001
During the puerperium, from 43 days to less than 1 year	−0.907–−0.887	<0.001
Not during pregnancy or postpartum period	−0.013–−0.000	0.0163
Inconsistent reported period	0.006–0.008	<0.001
Not informed or ignored	0.044–0.057	<0.001
**Type of maternal death**		
Direct obstetric maternal death	0.610–0.622	<0.001
Indirect obstetric maternal death	0.347–0.359	<0.001
Unspecified obstetric maternal death	0.028–0.033	<0.001
**Status of the death investigation**		
Death investigated, with summary record provided	−0.038–−0.018	<0.001
Death investigated, without summary record provided	−0.007–0.005	0.7275
Death not investigated	0.021–0.037	<0.001

**Source:** Prepared by the authors. Brazil, 2025.

## Data Availability

The original contributions presented in this study are included in the article. Additional questions can be directed to the corresponding author.

## Abbreviations

ICD-10	Classification of Diseases–10th Revision
DATASUS	Departamento de Informática do Sistema Único de Saúde
GBD	Global Burden of Disease
MMR	Maternal Mortality Ratio
SIM	Sistema de Informação sobre Mortalidade
SINASC	Sistema de Informação sobre Nascidos Vivos
STROBE	Strengthening the Reporting of Observational Studies in Epidemiology
WHO	World Health Organization
